# Supplementary material for: Risk factors for postoperative ileus in hysterectomy: A systematic review and meta-analysis
Source: PLoS One. 2024 Aug 1;19(8):e0308175. doi: 10.1371/journal.pone.0308175 (PMC11293682; doi:10.1371/journal.pone.0308175)
Supplement: S1 File — (DOCX) [file pone.0308175.s002.docx]

**S1 Fig.** **Forest plot of the association between use Opioids and POI in hysterectomy.**

**
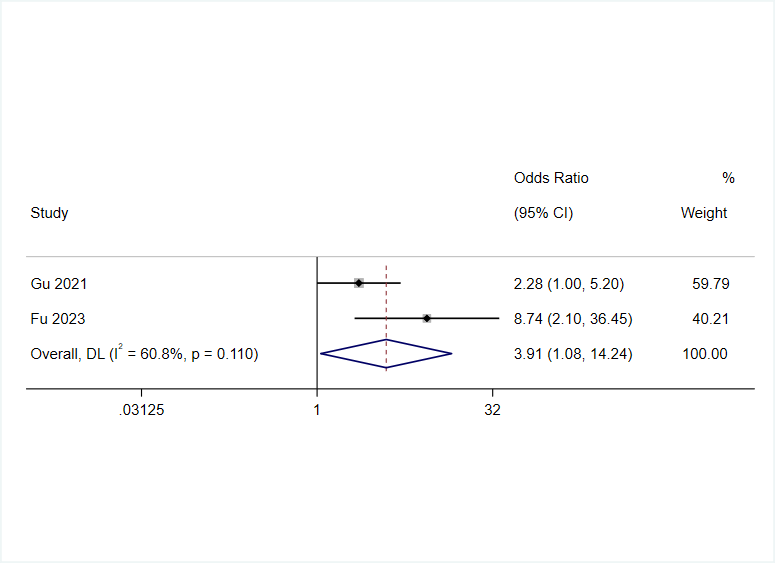
**

**S2 Fig. Forest plot of the association between dysmenorrhea and POI in hysterectomy.**

**
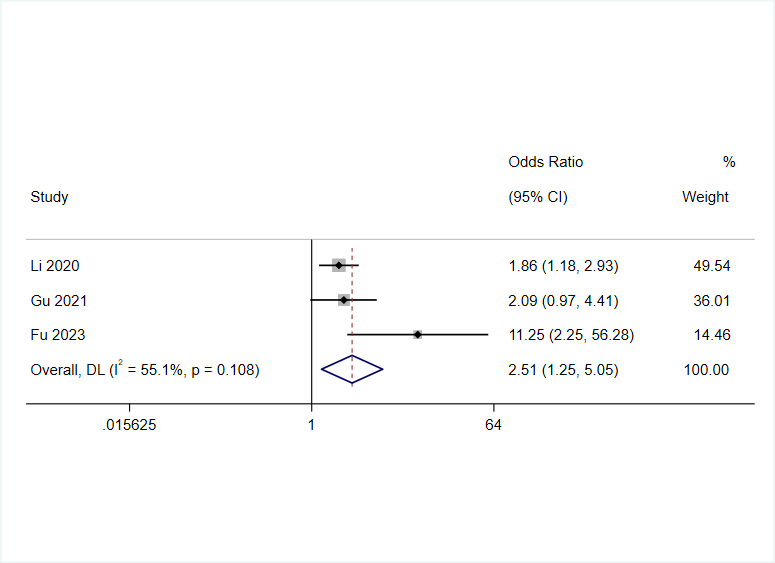
**

**S3 Fig. Forest plot of the association between smoking and POI in hysterectomy.**

**
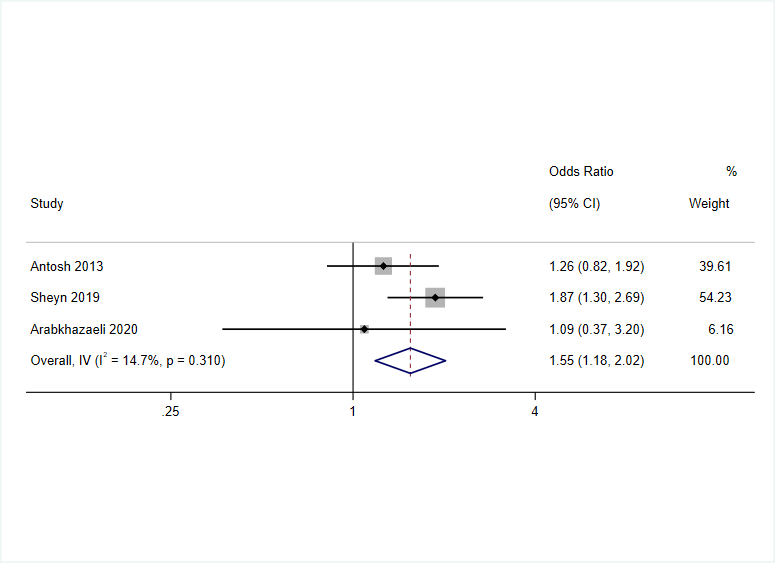
**

**S4 Fig. Forest plot of the association between prior abdominal or pelvic surgery and POI in hysterectomy.**

**
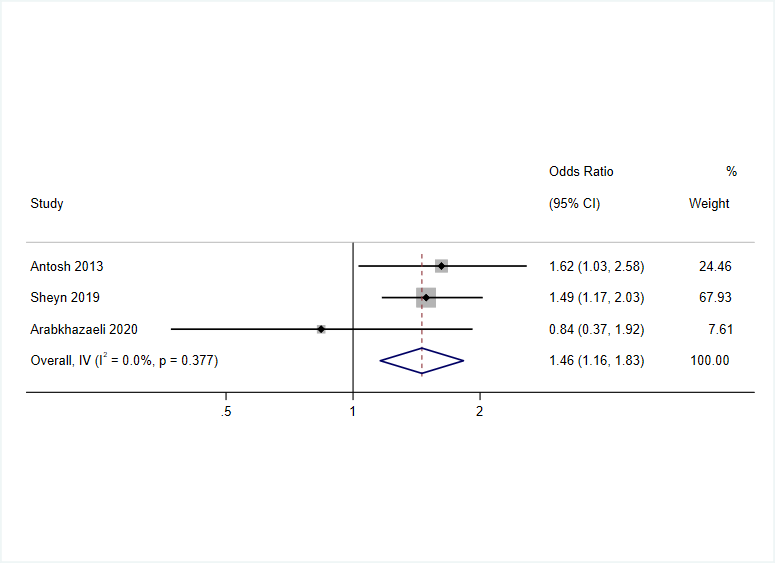
**

**S5 Fig. Forest plot of the association between age and POI in hysterectomy.**

**
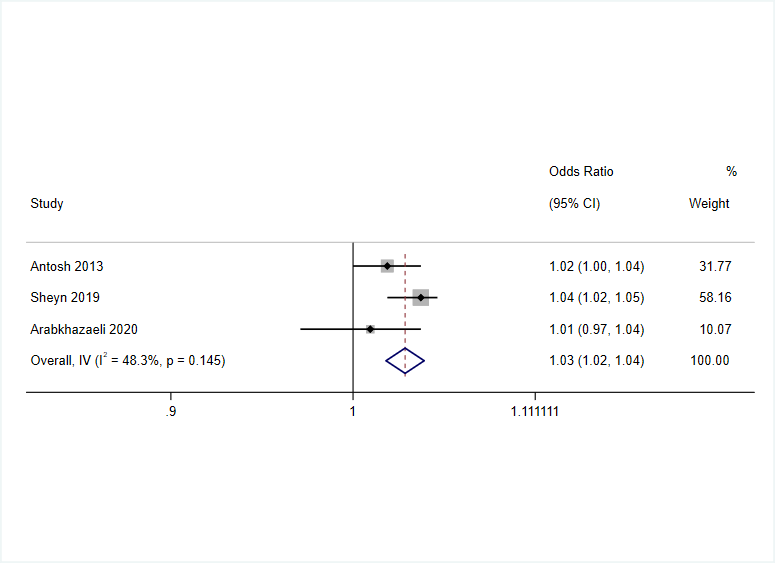
**

**S6 Fig. Forest plot of the association between perioperative transfusion and POI in hysterectomy.**

**
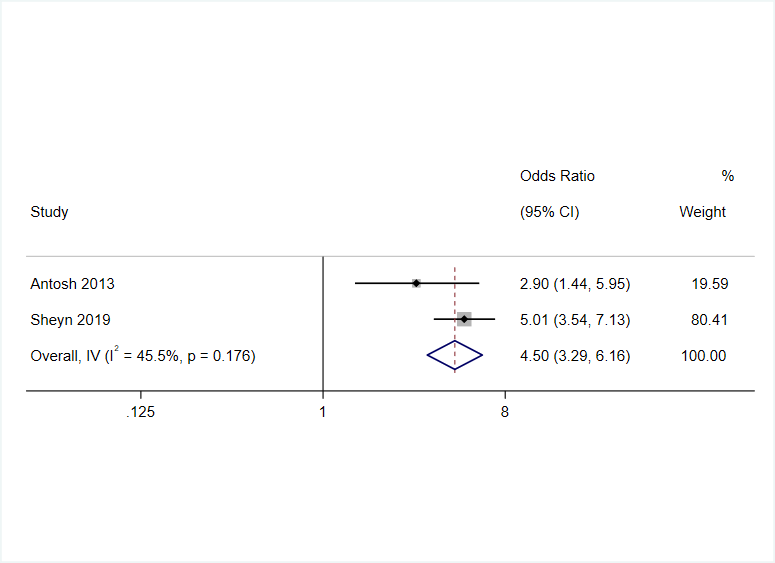
**

**S7 Fig. Forest plot of the association between concomitant bowel surgery and POI in hysterectomy.**

**
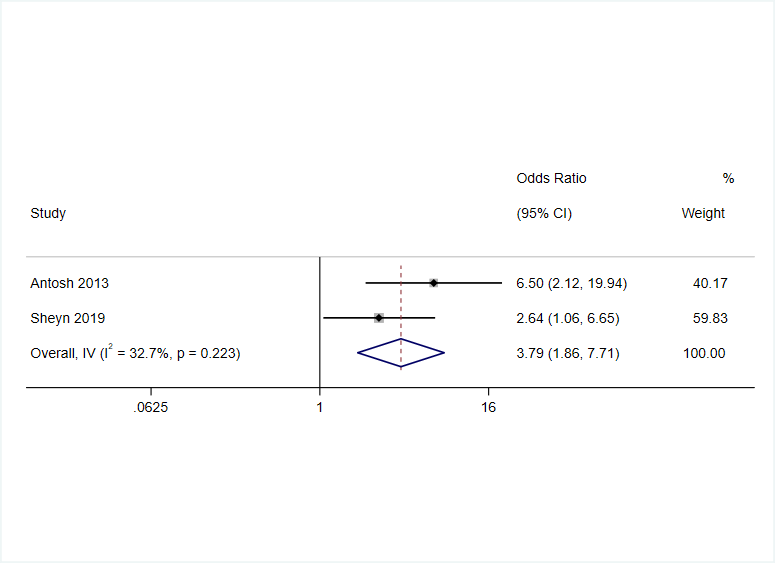
**

**S8 Fig. Forest plot of the association between anesthesia technique (general anesthesia) and POI in hysterectomy.**

**
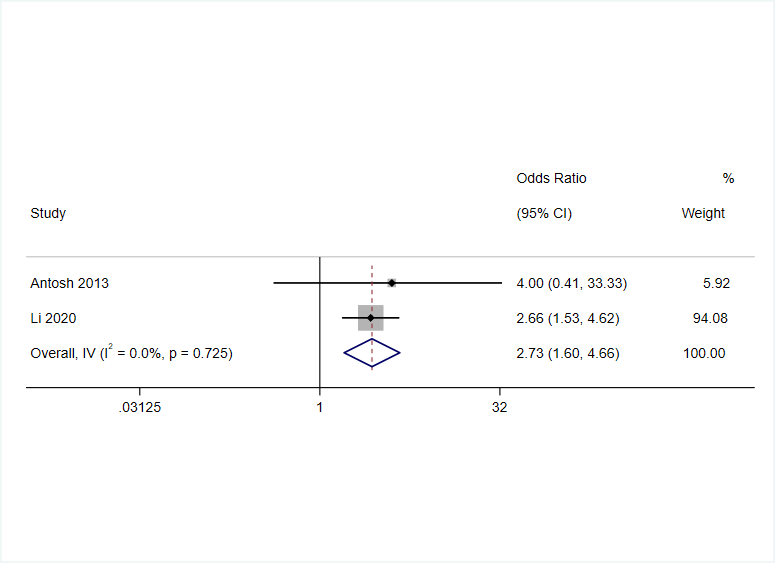
**

**S9 Fig. Forest plot of the association between adhesiolysis and POI in hysterectomy.**

**
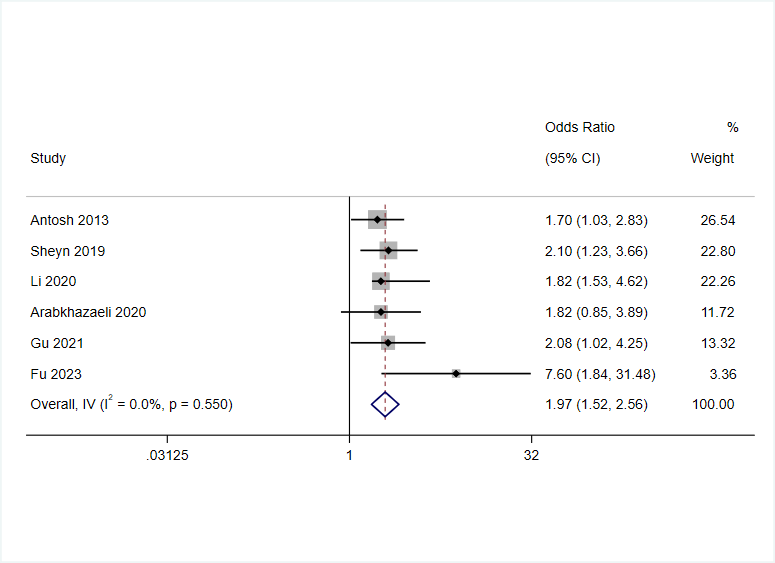
**

**S10 Fig. Forest plot of the association between duration of operation and POI in hysterectomy.**

**
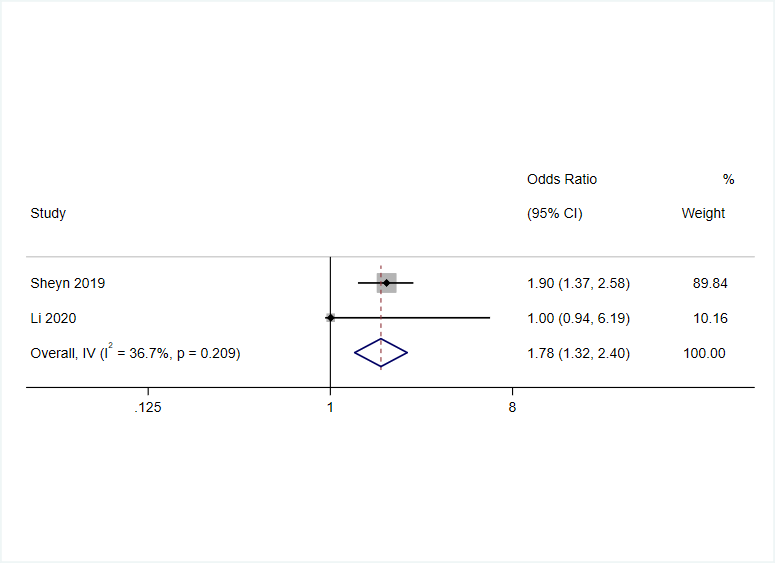
**

**S11 Fig. Forest plot of the association between operation approach (laparoscopic hysterectomy/vaginal hysterectomy) and POI in hysterectomy.**

**
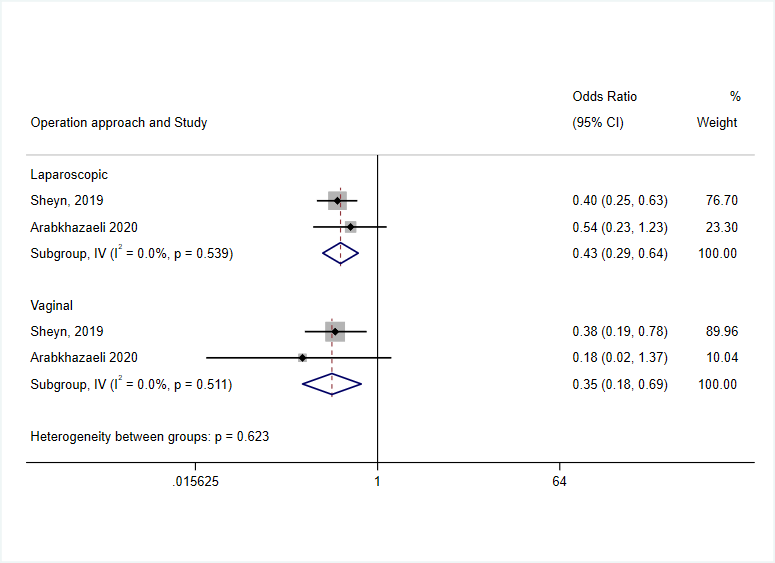
**

**S12 Fig. Forest plot of the association between BMI and POI in hysterectomy.**

**
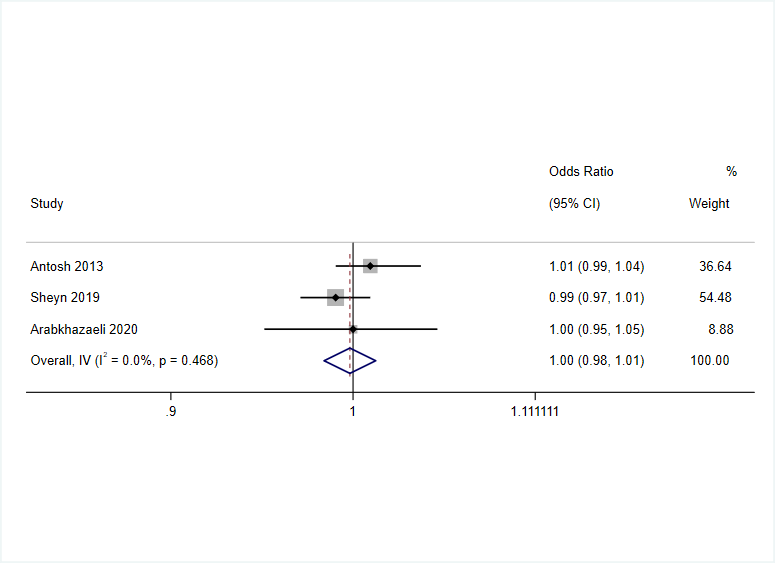
**

**S13 Fig. Forest plot of the association between diabetes and POI in hysterectomy.**

**
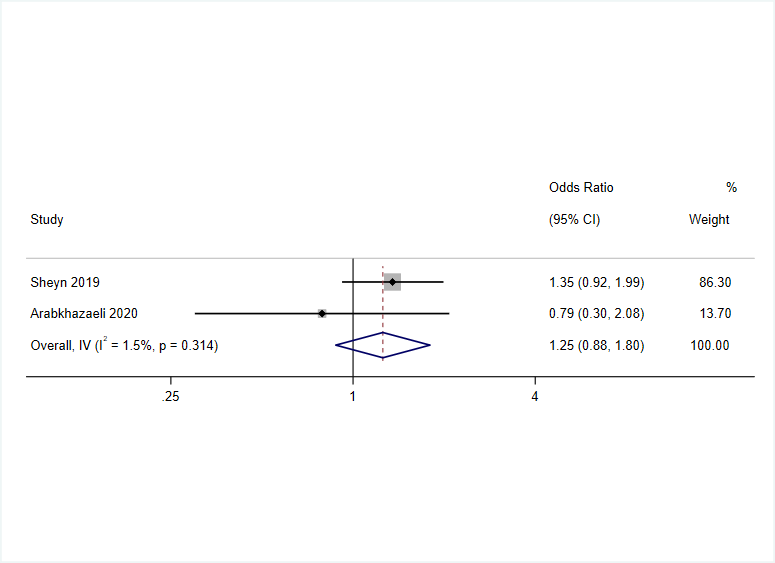
**

**S14 Fig. Forest plot of the association between endometriosis and POI in hysterectomy.**

**
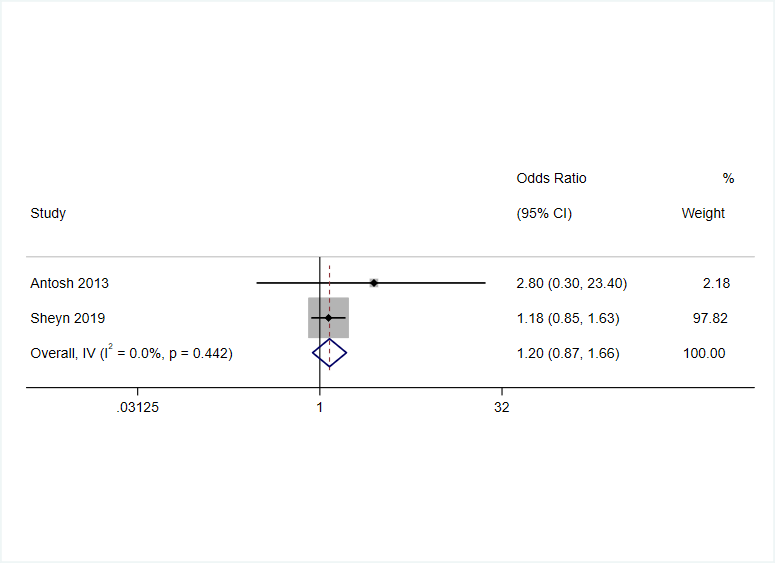
**

**S15 Fig. Forest plot of the association between nonwhite race and POI in hysterectomy.**

**
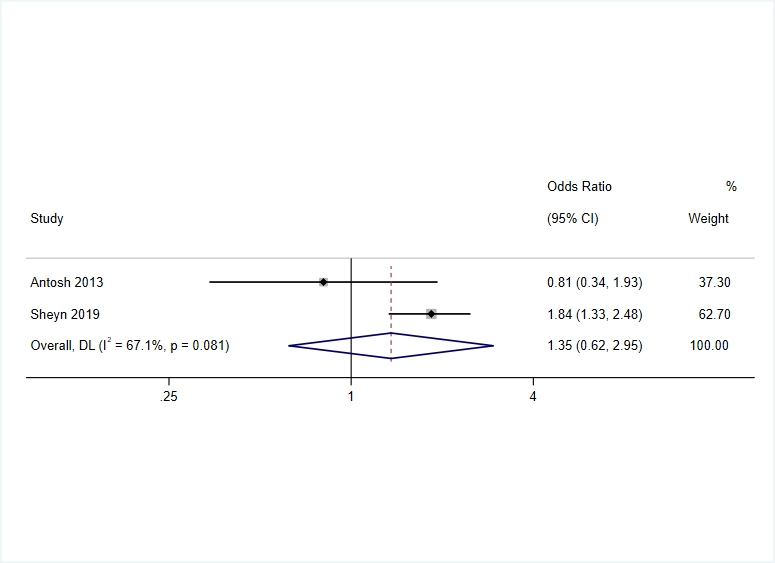
**
