# Supplementary material for: Risk factors for postoperative ileus in hysterectomy: A systematic review and meta-analysis
Source: PLoS One. 2024 Aug 1;19(8):e0308175. doi: 10.1371/journal.pone.0308175 (PMC11293682; doi:10.1371/journal.pone.0308175)
Supplement: S1 Table — (DOCX) [file pone.0308175.s003.docx]

**S1 Table. Search strategies in each database---2024.3.12**

| **Database** | **Search strategies** | **Results** |
| --- | --- | --- |
| **PubMed** | #1 "hysterectomy"[Mesh] ---34,250  #2 hysterectomy[Title/Abstract]---42,399  #3 #1 OR #2---54,715  #4 postoperative ileus[Title/Abstract] OR postoperative intestinal obstruction[Title/Abstract] OR ileus[Title/Abstract] OR intestinal obstruction[Title/Abstract]---29,494  #5 #3 AND #4---344 | 344 |
| **Web of Science** | #1 ((TS=(hysterectomy)) OR TI=(hysterectomy)) OR AB=(hysterectomy)---70,515  #2 TI=(postoperative ileus OR postoperative intestinal obstruction OR ileus OR intestinal obstruction) OR AB=(postoperative ileus OR postoperative intestinal obstruction OR ileus OR intestinal obstruction)---35,324  #3 #1 AND #2---437 | 437 |
| **Embase** | #1 'hysterectomy'/exp OR hysterectomy:ti,ab,kw---106,226  #2 'postoperative ileus':ti,ab,kw OR 'postoperative intestinal obstruction':ti,ab,kw OR ileus:ti,ab,kw OR 'intestinal obstruction':ti,ab,kw---38,883  #3 #1 AND #2---727 | 727 |
| **Cochrane library** | #1 (hysterectomy):ti,ab,kw (Word variations have been searched)---8,822  #2 (postoperative ileus OR postoperative intestinal obstruction OR ileus OR intestinal obstruction):ti,ab,kw (Word variations have been searched)---4,551  #3 #1 AND #2---130 | 130 |
| **CNKI** | #1 (SU=hysterectomy) OR (TKA=hysterectomy)---23,700  #2 (TKA=postoperative ileus OR postoperative intestinal obstruction OR ileus OR intestinal obstruction)---411  #3 #1 AND #2---6 | 6 |
